# Butyrate produced by gut commensal bacteria activates *TGF-beta1* expression through the transcription factor SP1 in human intestinal epithelial cells

**DOI:** 10.1038/s41598-018-28048-y

**Published:** 2018-06-27

**Authors:** Camille Martin-Gallausiaux, Fabienne Béguet-Crespel, Ludovica Marinelli, Alexandre Jamet, Florence Ledue, Hervé M. Blottière, Nicolas Lapaque

**Affiliations:** 1 Micalis Institute, INRA, AgroParisTech, Université Paris-Saclay, 78350 Jouy-en-Josas, France; 2 Sorbonne Université, Collège Doctoral, F-75005 Paris, France; 3 MetaGenoPolis, INRA, Université Paris-Saclay, 78350 Jouy en Josas, France

## Abstract

The intestinal microbiota contributes to the global wellbeing of their host by their fundamental role in the induction and maintenance of a healthy immune system. Commensal bacteria shape the mucosal immune system by influencing the proportion and the activation state of anti-inflammatory regulatory T cells (Treg) by metabolites that are still only partially unravelled. Microbiota members such as *Clostridiales* provide a transforming growth factor β (TGFβ)-rich environment that promotes the accumulation of Treg cells in the gut. The intestinal epithelial cells (IECs) take a central part in this process, as they are a major source of TGFβ1 upon bacterial colonisation. In this study, we investigated which gut commensal bacteria were able to regulate the *TGFB1* human promoter in IECs using supernatants from cultured bacteria. We reported that Firmicutes and Fusobacteria supernatants were the most potent *TGFB1* modulators in HT-29 cells. Furthermore, we demonstrated that butyrate was the main metabolite in bacterial supernatants accounting for TGFβ1 increase. This butyrate-driven effect was independent of the G-protein coupled receptors GPR41, GPR43 and GPR109a, the transporter MCT1 as well as the transcription factors NF-κB and AP-1 present on *TGFB1* promoter. Interestingly, HDAC inhibitors were inducing a similar *TGFB1* increase suggesting that butyrate acted through its HDAC inhibitor properties. Finally, our results showed that SP1 was the main transcription factor mediating the HDAC inhibitor effect of butyrate on *TGFB1* expression. This is, to our knowledge, the first characterisation of the mechanisms underlying *TGFB1* regulation in IEC by commensal bacteria derived butyrate.

## Introduction

Humans are colonized by bacteria, archaea, eukaryotes and viruses, which are collectively called the microbiota. These organisms exert diverse functions often associated with beneficial physiological effects for the host. Numerous studies suggest that indigenous members of the microbiota are central for the fine regulation of the host immune system through their intimate interaction with the host epithelium^1–5^. The composition of the host intestinal microbiota shapes the mucosal and the systemic immune responses in part by influencing the proportion and the activation state of T cell subsets. Among them the intestinal Foxp3^+^ regulatory T cells (Treg) play a central role in maintaining immunological homeostasis and their proportion is highly related to the microbiota composition^4,6–9^. Two studies showed that complex mixtures of *Clostridiales* bacteria from clusters IV and XIVa or a single *Clostridium* species cluster I, provide a transforming growth factors β1 (TGFβ1)-rich environment that promotes the accumulation of induced Treg cells (iTreg)^4,9^. Interestingly, in inflammatory bowel diseases (IBD) known to be linked to commensal bacteria composition, *Clostridiales* clusters IV and XIVa are significantly less abundant and the pathology have been reported to be associated with an impairment of TGFβ signalling in numerous immune cell types including T cells^10–16^.

The molecular mechanisms of Treg cell generation by the microbiota have only been partially unravelled^4,6–9^. Several groups have shown that short chain fatty acids (SCFAs), such as butyrate, that derived from the bacterial fermentation of dietary fibres promote Treg cell generation^6,8^. Butyrate acts as an inhibitor of histone deacetylases (iHDACs) and consequently enhances histone H3 acetylation in the regulatory elements of the *Foxp3* locus. However, *in vitro* Treg generation is not induced directly by SCFAs alone and requires TGFβ1 stimulation^6,8^. Moreover, TGFβ1 is essential for Treg differentiation by butyrate-producing bacteria *in vivo*^4^.

In the gut, dendritic cells (DC) and intestinal epithelial cells (IEC) are the major cellular sources of TGFβ1^4,9,17–19^. Interestingly, germ-free mice colonisation by certain members of the microbiota leads to enhanced TGFβ1 expression in IECs and the almost complete restoration of colonic Treg level^4,9,20,21^. A recent study demonstrated that *Clostridium butyricum* increased iTreg generation *via* a Toll-like receptor 2 (TLR2)-dependent induction of TGFβ1 by DCs. This was the first demonstration of a mechanism deployed by a member of the gut microbiota to activate TGFβ1 secretion leading to Treg cells generation^9^.

The *TGFB1* auto-induction loop is well documented. Addition of TGFβ1 activates TGFβ1 receptor leading to a SMAD and MAPK-dependent downstream signalling^22^. However, there are only few mechanical insights on *TGFB1* regulation by exogenous signals. Indeed, TGFβ1 induction by commensal bacteria is promoting the proliferation, polarization and differentiation of T lymphocytes but the involved molecules or pathways are still unknown^4^. We thus decided to investigate the impact of individual cultivable commensal bacteria on *TGFB1* transcriptional expression in a human IEC model and to further characterise the underlying molecular mechanisms.

In the current study, we screened bacterial supernatants derived from over 120 commensal species on a *TGFB1* reporter system and showed that butyrate was the main microbiota-derived metabolite inducing *TGFB1* expression in the human intestinal epithelial cell-line HT-29. We showed that the *TGFB1* induction by butyrate was independent of the SCFA G-protein coupled receptors (GPR41, GPR43 and GPR109a), and of the SCFA transporter monocarboxylate transporter 1 (MCT1). Moreover, the *TGFB1* up-regulation could be attributed to the HDAC inhibitory properties of SCFAs. Finally, by using specific inhibitors and point mutations of the promoter region of *TGFB1*, we excluded NFκB and AP1 as regulatory elements and showed that the SP1 transcription factor was involved in the butyrate-driven activation of *TGFB1* expression.

## Results

### Metabolites derived from commensal bacteria modulate TGFβ1 expression

**Figure 1:**
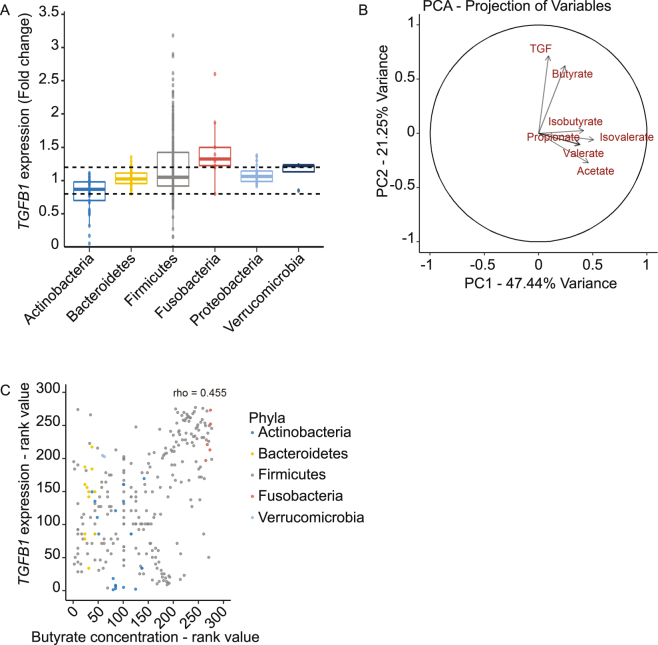
Correlation between bacterial metabolites production and *TGFB1* gene expression. (**A**) Effect of bacterial supernatants on HT-29-TGF_prom_ reporter system organised by phylum. Culture supernatants of a wide range of commensal bacteria were applied on the HT-29-TGF_prom_ reporter system (10% vol/vol). *TGFB1* expression was measured by luciferase activity and expressed as fold change towards its control: bacterial growth medium used in each experiment and bacterial culture. Dash lines draw upper and lower zones where *TGFB1* was consider as significantly changed. (**B**) PCA analysis showing the correlation between the SCFA concentrations produced the commensal bacteria and the *TGFB1* expression. (**C**) Representation of *TGFB1* expression correlated to butyrate concentration in different bacterial cultures ordered ranked values (Spearman correlation, r = 0.455, p-value = 1.302e-15). Actinobacteria in blue, Bacteroidetes in yellow, Firmicutes in grey, Fusobacteria in red and Verrucomicrobiea in light blue.

TGFβ1 expression is severely decreased in the colon of germ-free mice compared to colonised mice suggesting a crucial role of the microbiota in this process^4^. The impact of bacterial metabolites is particularly drastic on TGFβ1 production by IEC although no mechanism has been described at the moment. Thus, to decipher which commensal bacteria regulate *TGFB1* expression; we have studied the activity of more than a hundred bacterial species, including 60% of the bacteria belonging to the human core microbiota, on a *TGFB1* reporter system expressed in the human IEC line HT-29^23^. Previous publications have reported that active biological compounds produced by bacteria in the gut are likely to be small diffusible molecules, thus we tested bacterial supernatants on a HT-29-TGF_prom_ reporter system (Fig. 1A)^11,24–26^. Our results showed that species belonging to the Firmicutes and Fusobacteria phyla were the main *TGFB1* inducers while some members of Actinobacteria were inhibitors. At the genus level, *Clostridiales* and *Fusobacterium* were highly increasing *TGFB1* expression. *Lactobacillus* and *Bifidobacterium* were decreasing *TGFB1* activity however, these effects were not found in all the tested species (Supplementary Fig. 1).

### Butyrate activates *TGFB1* expression in epithelial cell line

A common characteristic of the main *TGFB1* inducers, *Clostridiales* and *Fusobacterium*, is their capacity to degrade complex dietary fibres by anaerobic fermentation^27^. As SCFAs are major end-products of this anaerobic fermentation with important impacts on human health, we thus quantified the concentrations of acetate, butyrate, propionate, valerate as well as isobutyrate and isovalerate in bacterial supernatants (Supplementary Table 1). Global correlation analysis by principal component (PCA) of all the SCFAs concentrations and *TGFB1* expression showed that butyrate was the only SCFA for which the concentration correlated to *TGFB1* activity (Fig. 1B). In addition, this association was confirmed by a Spearman pairwise correlation between butyrate and *TGFB1*, on ranked data (Fig. 1C).

**Figure 2:**
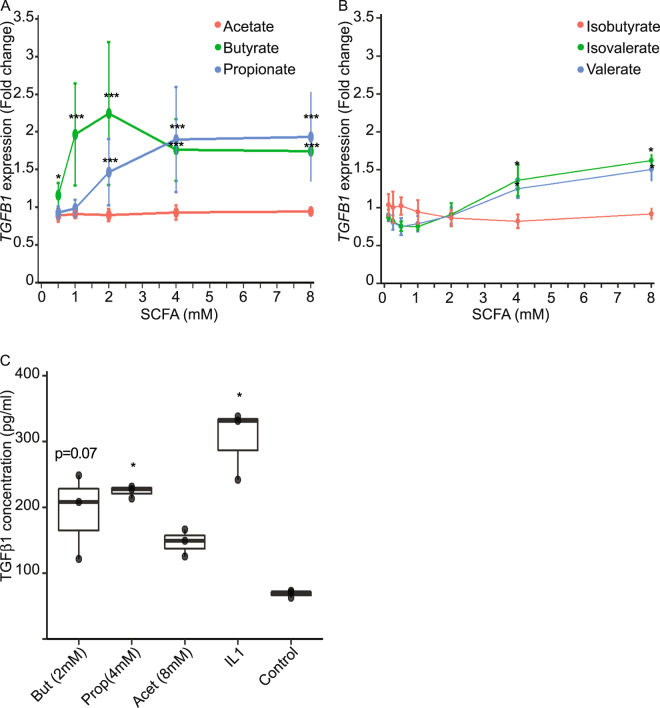
Impact of SCFAs on *TGFB1* expression. (**A**) HT-29-TGF_prom_ reporter cells were incubated for 24 h with a range of concentrations of acetate, propionate and butyrate (0.5, 1, 2, 4, 8 mM). (**B**) HT-29-TGF_prom_ reporter cells were incubated for 24 h with a range of concentrations of isobutyrate, isovalerate and valerate (0.5, 1, 2, 4, 8 mM). *TGFB1* expression was measured by luciferase activity and expressed as the mean ± SD of fold change towards un-stimulated cells, N ≥ 3, (**C**) HT-29 cells were incubated for 24 h with IL1β (10 ng/ml), butyrate 2 mM, propionate 4 mM or acetate 8 mM. TGFβ1 active and latent levels were measured by ELISA and expressed as median ± quartiles, expressed in pg/ml. N = 3, t-test, *P < 0.05, **P < 0.01, ***P < 0.001.

We confirmed experimentally the observed correlation by testing a large range of physiological concentrations of SCFAs on HT-29-TGF_prom_ reporter cells. As shown in Fig. 2A, butyrate was a potent *TGFB1* inducer with a significant impact at concentration starting at 1 mM. Interestingly, other SCFAs such as propionate, valerate and isovalerate activated *TGFB1* but to a lesser extent (Fig. 2A,B). The most abundant SCFA produced by gut bacteria, acetate, had no impact on *TGFB1* expression. Moreover, we demonstrated that SCFAs not only increased *TGFB1* gene activity but also enhance TGFβ1 protein level as assayed by ELISA (Fig. 2C).

### Butyrate-driven activation of *TGFB1* is independent of the SCFA receptors GPR41, GPR43, GPR109a and the SCFA transporter MCT1

**Figure 3:**
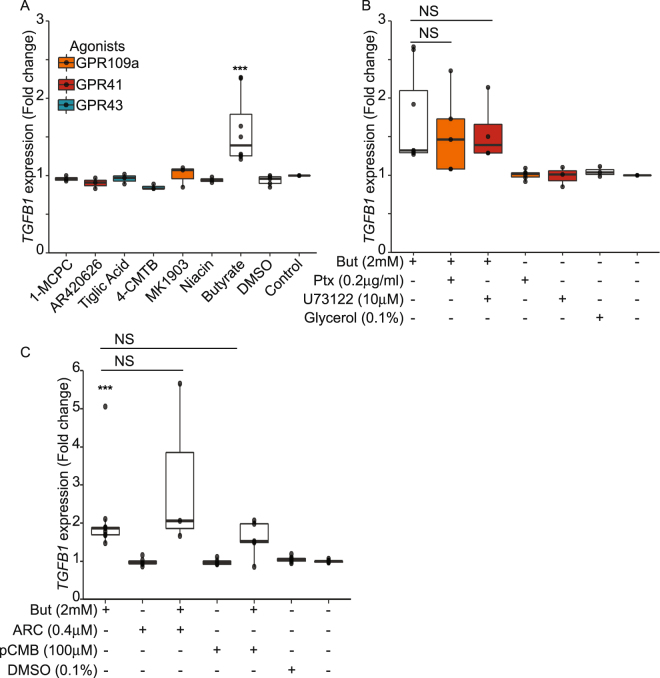
Butyrate mediated impact on *TGFB1* is independent of GPR41, GPR43, GPR109a receptors and MCT1 transporter. (**A**) HT-29-TGF_prom_ reporter cells were incubated for 24 h with selective GPR agonists: GPR41: AR420626 (1 μM) and 1-MCPC (1 mM); GPR43: 4-CMTB (1 μM) and Tiglic acid (1 mM); GPR109a: Niacin (1 mM) and MK1903 (1 μM) or with DMSO (vehicle) or butyrate (2 mM). (**B**) HT-29-TGF_prom_ reporter cells were incubated with GPRs sub-unit inhibitors: Pertussis toxin 2 h before (Ptx, 0.2 μg/ml) and U73122 (10 μM) prior butyrate 2 mM stimulation and left for a total 24 h of incubation. (**C**), HT-29-TGF_prom_ reporter cells were incubated for 24 h with selective MCT1 inhibitors: pCMB (100 μM) and AR-C155858 (400 nM),. *TGFB1* expression was measured by luciferase activity and expressed as median ± quartiles of fold change towards un-stimulated cells. N ≥ 3, Wilcoxon test, *P < 0.05, **P < 0.01, ***P < 0.001.

SCFAs induce numerous signalling pathways through their binding to three different G-protein coupled receptors (GPR): GPR41, GPR43 and GPR109a. These receptors display affinities for SCFAs leading to cellular signals^28–30^. The three GPRs were found expressed in HT-29 cell line (Supplementary Fig. 2A). To investigate the potential role of these receptors on *TGFB1* expression, we tested specific agonists of GPR41 (1-MCPC and AR420626), GPR43 (Tiglic acid and 4-CMTB) and GPR109a (Niacin and MK1903) on HT-29-TGF_prom_ reporter system. None of the agonists was able to mimic the effect of butyrate on *TGFB1* expression suggesting that these GPRs were not involved in this process (Fig. 3A). Upon ligand binding, GPRs signal through two G-protein subunits activation: Gα/i (for GPR41, GPR43 and GPR109a) and Gα/q (for GPR43). To confirm our observation, we used Gi/o and Gq inhibitors, the pertussis toxin (Ptx) and phospholipase Cβ inhibitor (U73122) respectively. Interestingly, none of the inhibitors was able to block the butyrate-dependent up-regulation of *TGFB1* expression (Fig. 3B). Moreover, over-expression of GPR43 and GPR109a in HT-29-TGF_prom_ reporter cells did not impact the butyrate-dependent activation of *TGFB1* expression, strengthening the hypothesis of a GPR-independent mechanism (Supplementary Fig. 2B and C).

Monocarboxylate transporter 1 (MCT1) has been described as the main butyrate transporter in the colon and is largely expressed in HT-29 cells (Supplementary Fig. 2A)^31,32^. We thus investigated if butyrate uptake by monocarboxylate transporter 1 (MCT1) was involved in *TGFB1* up-regulation. Incubation of HT-29-TGF_prom_ cells with two different MCT1 inhibitors, pCMB and AR-C15858, had no impact on the butyrate-induced activation of *TGFB1*, suggesting that MCT1 was not involved in the butyrate-driven up-regulation of *TGFB1* (Fig. 3C).

### Butyrate induces *TGFB1* expression via its HDAC inhibitor property

**Figure 4:**
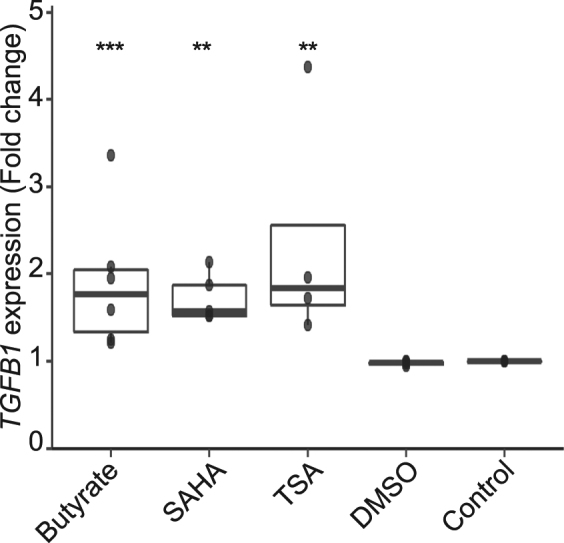
HDAC inhibitor mimicked the butyrate-dependent activation of *TGFB1* expression. HT-29-TGF_prom_ reporter cells were incubated with butyrate (2 mM), SAHA (5 μM), trichostatin A, (TSA, 1 μM) or controls (DMSO and RPMI). *TGFB1* expression was measured by luciferase activity and expressed as fold increase towards the control: un-stimulated cells. Data are represented as median ± quartiles of fold change towards un-stimulated cells. N ≥ 3, Wilcoxon test, *P < 0.05, **P < 0.01, ***P < 0.001.

It is well established that SCFAs, and butyrate in particular, rely on their lysine and histone deacetylase inhibition (HDACi) property to modulate histone acetylation, transcription factor acetylation and binding, and consequently gene expression^5,33–35^. Butyrate targets class I and class II HDAC and thus modulates the expression of a wide range of genes^36^. To asses if butyrate HDACi property was involved in *TGFB1* up-regulation, we tested two HDACi targeting zinc dependent class I and II HDAC families. We treated HT-29-TGF_prom_ reporter cells with trichostatin A (TSA) and vorinostat (SAHA) both sharing a hydroxynamic acid structure, different from butyrate which belongs to the aliphatic family^33,36^. Interestingly, both TSA and SAHA mimicked butyrate effect by increasing *TGFB1* activity, suggesting that the butyrate-driven up-regulation of *TGFB1* might be a consequence of its HDAC inhibitory properties (Fig. 4).

### Butyrate-induced *TGFB1* expression is independent of NF-κB and AP-1 and relies on SP1

Regulation of gene transcription by butyrate involves a wide range of transcription factors. To delineate which transcription factors (TF) are targeted by butyrate and might directly impact *TGFB1* expression, we analysed the human *TGFB1* promoter sequence. As expected from previous publications, we found several binding sites for transcription factors implicated in HDACi and butyrate-regulated gene expression including Specificity Protein-1 (SP1) binding GC-rich boxes, Activator protein 1 (AP-1) and NF-κB responsive elements (Table 1)^37–45^. Previous work reported that some of these binding sites were functional. Indeed an AP-1 and a SP1 binding sites have been described as main regulators of the *TGFB1* gene activity in different cell types at steady state and upon infection^46,47^. NF-κB was shown to increase *TGFB1* expression in cooperation with AP-1^48^.

**Table 1:** Selection of transcription factor binding motifs in human *TGFB1* promoter including localisation of AP-1 and SP1 mutations.

Transcription Factor	Positions	Mutations	References
AP-1	−820; − 418; −371; +159	−416/−415; −371/−370	Kim *et al*.^38^, Lee *et al*.^48^, Weigert *et al*.^46^
SP1	−236; −220; −120; −108; −79; −43; +175	−216/−215	Kim *et al*.^38^, Lee *et al*.^48^, Weigert *et al*.^46^
NF-κB	−1267; −1174; +52		Lee *et al*.^48^

**Figure 5:**
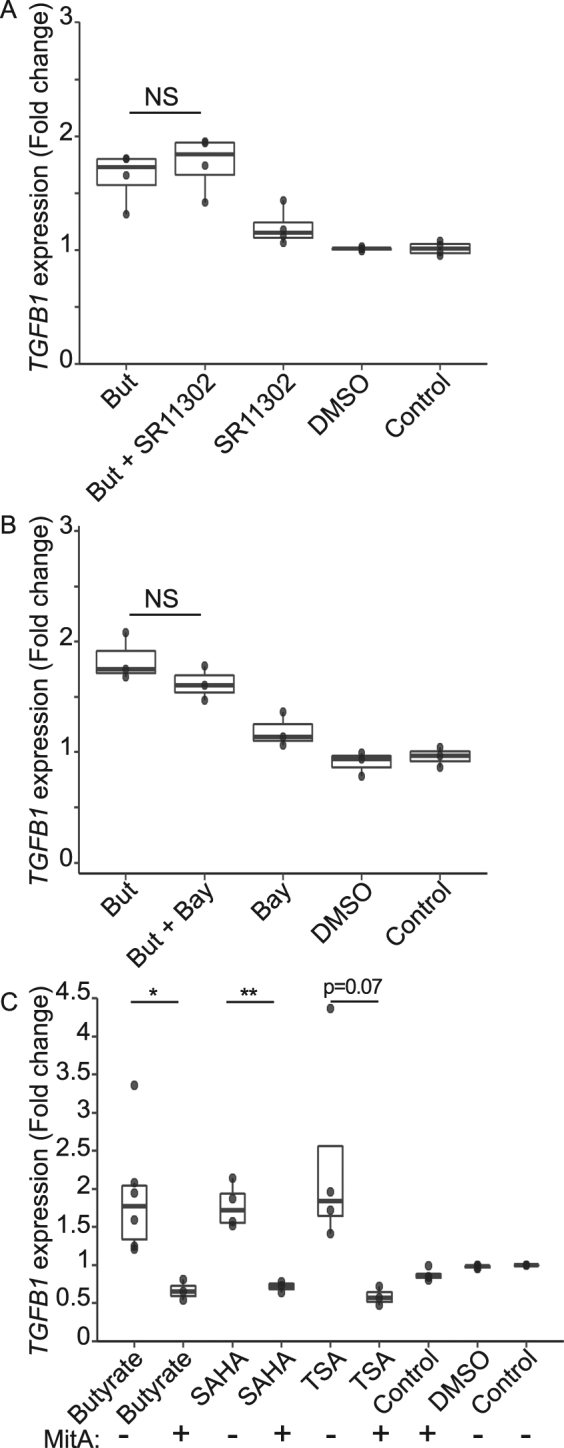
Butyrate activated *TGFB1* expression in a NF-κB-independent, AP1-independent-manner. (**A**) HT-29-TGF_prom_ reporter cells were stimulated for 24 h with SR11302 (10 µM) a specific AP-1 inhibitor in presence or absence of butyrate (But 2 mM). Wilcoxon test,, N = 4; (**B**) HT-29-TGF_prom_ cells were incubated with BAY11-7082 40 µM 2 h prior incubation with butyrate (But 2 mM) or controls. T-test, N = 3; (**C**) HT-29-TGF_prom_ cells were incubated for 24 h with mithramycin A (100 nM) and butyrate or HDACi, t-test, N ≥ 3. *TGFB1* expression was measured by luciferase activity and expressed as fold increase towards un-stimulated cells. Data are represented as median ± quartiles of fold change. *P < 0.05, **P < 0.01, ***P < 0.001.

To investigate which transcription factor controls the butyrate-induced up-regulation of *TGFB1*, we first treated HT-29-TGF_prom_ reporter cells with an AP-1 specific inhibitor (SR11302) in presence or absence of butyrate (Fig. 5A). The AP-1 inhibitor was not able to block the butyrate-induced *TGFB1* increase suggesting that this transcription factor was not involved. Then, we treated HT-29-TGF_prom_ reporter cells with an NF-κB inhibitor (BAY 117082) prior to butyrate stimulation (Fig. 5B). We observed that NF-κB inhibition did not impact significantly butyrate enhancement of *TGFB1*. Interestingly, butyrate did not activate the NF-κB pathway in a HT-29 NF-κB reporter system confirming that this transcription factor had no role in butyrate-dependent *TGFB1* induction (Supplementary Fig. 3A). To explore SP1 implication in the induction of *TGFB1* in our model, we incubated HT-29-TGF_prom_ reporter cells with butyrate or HDACi in presence of mithramycin A, a competitive SP1/SP3 inhibitor. Binding inhibition to GC rich sequence by mithramycin A drastically inhibited *TGFB1* up-regulation by butyrate (Fig. 5C). We also observed a diminution of the butyrate-dependent activation of *TGFB1* by inhibiting SP1 expression by siRNA thus strengthening our previous result (Supplementary Fig. 3B). These results suggest that the induction of *TGFB1* expression by butyrate is mediated through SP1 binding to GC rich response element. Furthermore, TSA and SAHA impacts on *TGFB1* expression were also abrogated by SP1 knockdown or mithramycin A competition (Fig. 5C and Supplementary Fig. 3B).

**Figure 6:**
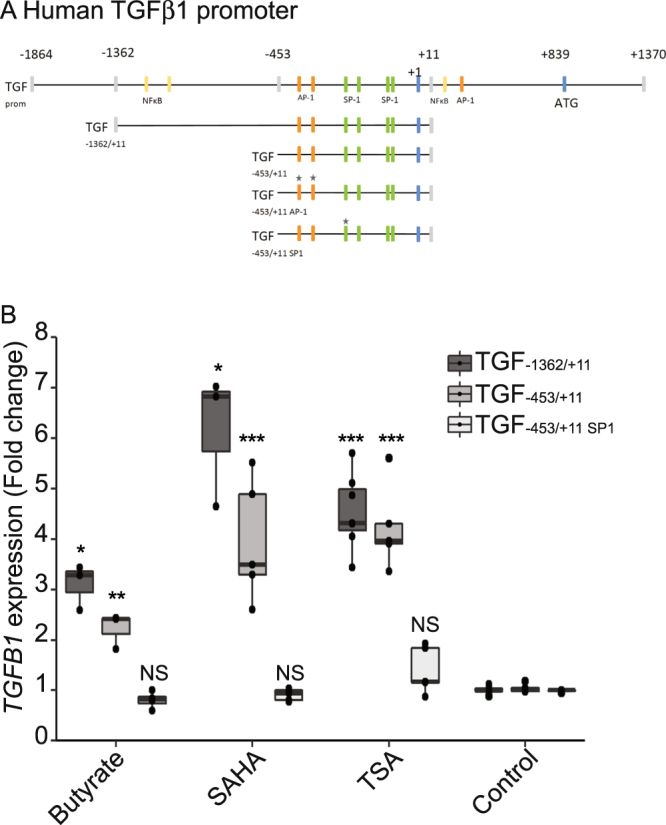
*TGFB1* up-regulation by butyrate and HDACi is SP1-dependent (**A**) Schematic representation of the human *TGFB1* promoter with a selection of binding motifs^37,38,46,47^. The translation (ATG) and transcription start sites (+1) were defined by^65,66^. Mutations of AP-1 and SP1 boxes on HT-29-TGF_−453/+11_ are represented. (**B**) HT-29-TGF_−1362/+11_, HT-29-TGF_−453/+11_, HT-29-TGF_−453/+11SP1_ reporter cells were stimulated for 24 h with butyrate (4 mM), TSA (1 µM) or SAHA (5 µM). *TGFB1* expression was measured by luciferase activity and expressed as fold increase towards un-stimulated cells. Data are represented as median ± quartiles of fold change. N ≥ 3, t-test, *P < 0.05, **P < 0.01, ***P < 0.001.

We further characterised which region of *TGFB1* promoter was responsible for the butyrate dependent activation of *TGFB1*. Two truncated *TGFB1* promoter constructs were generated and stably transfected into HT-29 cells. HT-29-TGF_−1362/+11_ and HT-29-TGF_−453/+11_ reporter cells were treated with butyrate and HDAC inhibitors (Fig. 6A,B). The effect of butyrate and HDACi on luciferase expression was observed in both constructs suggesting a cis-regulatory element activity present in the core promoter, close to the 5′ UTR region (Fig. 6B).

As shown in Fig. 6A, several functional AP-1 (−416, −370) and SP1 (−216) responsive elements have been identified in the *TGFB1* core promoter^46,47^. To assess the role of the AP-1 (−416, −370) and SP1 (−216) binding sites, we stably transfected HT-29 cells with the TGF_−453/+11_ reporter system with a double mutations of the AP-1 motifs or a single mutation on the SP1 motif (HT-29-TGF_−453/+11AP1_ and HT-29-TGF_−453/+11SP1_ respectively, Fig. 6A). Mutations of the AP-1 motifs did not prevent the activation by butyrate and others HDACi confirming our results with AP-1 inhibitor (Supplementary Fig. 4). Mutation of one SP1 binding site was sufficient to severely reduce the *TGFB1* promoter activation by butyrate and HDACi, confirming that SP1 is involved in this process (Fig. 6B). Altogether our results showed that butyrate and HDACi induce *TGFB1* up-regulation independently of NF-κB and AP-1 and through the −216 SP1 site.

### Bacterial induction of *TGFB1* is butyrate and SP1-dependent

**Figure 7:**
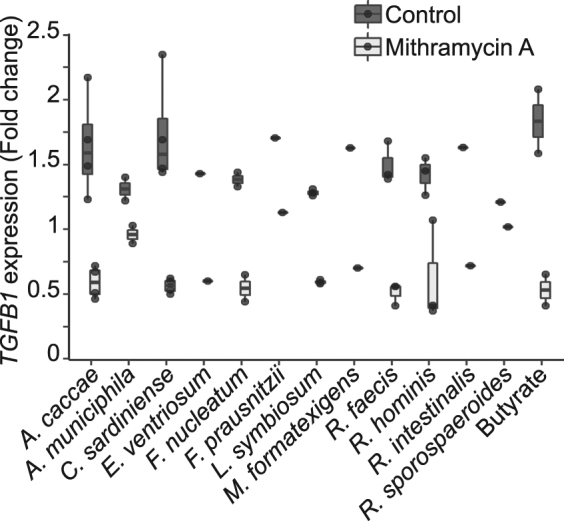
SP1-inhibitor mithramycin inhibited *TGFB1* induction by the butyrate-producing bacteria. HT-29-TGF_prom_ cells were incubated for 24 h with bacterial supernatants (10% (v/v)) or butyrate (But 2 mM) ± mithramycin A (100 nM). *TGFB1* expression was measured by luciferase activity and expressed as fold increase towards un-stimulated cells. Data are represented as median ± quartiles of fold change.

The main purpose of this study was to investigate the molecular mechanism by which commensal bacteria induce *TGFB1* expression in epithelial cells. We thus incubated a selection of *TGFB1*-inducing bacterial supernatants on HT-29-TGF_prom_ cells in presence of SP1/SP3 inhibitor (mithramycin A). As shown in Fig. 7, inhibition of SP1/SP3 binding motifs had a similar impact on butyrate and bacterial-induced up-regulation of *TGFB1*. The commensal bacteria selected throughout our screen due to their ability to increase *TGFB1*, were all butyrate-producing bacteria. Our results demonstrate the crucial role of SP1 in the *TGFB1* expression induced by butyrate from commensal bacteria.

## Discussion

TGFβ1 is a pleiotropic cytokine highly conserved among metazoan and secreted in its latent form. Produced by many cell and tissue types, TGFβ1 is involved in various biological functions including pluripotency, tissue morphogenesis, cellular differentiation and regeneration^49^. As a crucial cytokine of tissue homeostasis, TGFβ misregulations are involved in many pathologies such as cancer, fibrosis, asthma, infections and inflammatory diseases. More specifically, TGFβ1, the most abundant TGFβ isoform, plays complex roles in the gut immune response regulation^50^.

Several studies highlighted the importance of TGFβ1 as a negative regulator of mucosal inflammatory responses and showed that defects in its expression or signalling lead to colitis^4,9,13–15^. In human, *SMAD3* an intracellular molecule involved in the TGFβ signalling pathway is among the loci associated with high IBD susceptibility^22,51^. Moreover, defective TGFβ1 signalling in IBD patients has been associated with high SMAD7 expression, an inhibitor of SMAD2/3 phosphorylation. SMAD7 deleterious effects can be reversed by treatment with a SMAD7 antisense nucleotide^13–15^. Many studies demonstrated the involvement of gut microbiota in the development of the local and systemic immune system notably by the modification of regulatory T cell (Treg) differentiation^4,6–9^. In the gut, dendritic cells (DC) and intestinal epithelial cells (IECs) are the major cellular sources of TGFβ1 induced by the microbiota that is essential for the generation of Treg cells^4,9,17,20^. Although IECs are not the main TGFβ1 producers *per se*, yet they are an important source of this cytokine when considering the huge number of cells they represent in the gut^19^. Moreover, IECs are at the interface between the microbiota and the *lamina propria* and are thus more likely to be influenced by commensal bacteria in particular in making the gut a TGFβ1-rich environment^17,52^.

Here, we demonstrated that, amongst the microbial metabolites produced by the commensal bacteria tested, short chain fatty acids (SCFAs), and more specifically butyrate, had a major impact on human *TGFB1* expression in IECs (Fig. 1). We showed that physiological concentrations of butyrate up-regulated the expression of *TGFB1* in HT-29 (Fig. 2). Our findings confirmed previous studies showing that butyrate is a potent *TGFB1* inducer in diverse cell types including IECs^4,53–55^. We investigated the mechanism of butyrate-driven *TGFB1* induction. In assays using GPRs ligands, G-protein sub-units inhibitors and GPR over-expression, we established that butyrate impact on *TGFB1* was independent of GPR43, GPR41 and GPR109a. Similarly, inhibition of the MCT1 transporter did not abrogate butyrate effect, pointing out that butyrate uptake by MCT1 was dispensable (Fig. 3). Yet, MCT1 belongs to a large family of transporter (MCTs) mediating monocarboxylate uptake from gut lumen to cell cytoplasm. Among MCTs, MCT1 is the most expressed in the human large intestine but we cannot exclude that other expressed isoforms such as MCT4 may be involved in butyrate transport^56,57^. It is also possible that butyrate enters the cell *via* passive transport or diffusion^31,32,56,57^. SCFAs, particularly butyrate, impact the host biological responses by the direct regulation of gene transcription *via* their properties of histone deacetylase inhibitors (HDACi) that consequently favours acetylation of histones^5,34,35,58^. We demonstrate that two HDAC inhibitors structurally and metabolically unrelated to SCFAs were inducing *TGFB1* expression similarly to butyrate. Thus it is likely that the butyrate-induced *TGFB1* up-regulation was a consequence of its HDAC inhibitory properties. To our knowledge up-regulation of *TGFB1* expression by HDACi has never been reported, especially in IECs.

Regulation of gene transcription by HDACi involves a wide range of transcription factors that can bind *TGFB1* promoter and that includes NF-κB, AP-1 and SP1^40,42–45^. Several responsive elements of these transcription factors were shown to be involved in *TGFB1* induction by different stimuli. Indeed, it has been described that immune signals such as IL1β, TNFα and Toll-like receptors ligands can activate *TGFB1* transcription through NF-κB activation^9,48^. Two AP-1 motifs were shown to be functional in hepatic and mesangial cells and confirmed in our study by using phorbol ester^46,47,59^ (Supplementary Fig. 4). Moreover, previous publications demonstrated that the Specific protein 1 (SP1) binding motifs are particularly enriched in *TGFB1* promoter and that one of them modulates *TGFB1* expression in various models, at steady state and upon infection. We investigated the role of these transcription factors in *TGFB1* up-regulation by butyrate. NF-κB and AP-1 inhibitors did not impact on butyrate-driven up-regulation of *TGFB1* suggesting that these two transcription factors were not involved. These results were confirmed by the fact that butyrate did not activate a NF-κB reporter system in HT-29 and that a *TGFB1* promoter construct mutated for the 2 functional AP-1 motifs responded to butyrate similarly to the wild-type *TGFB1* promoter. *TGFB1* promoter has a high number of GC-rich motifs that are potential targets for the transcription factors SP1 and SP3. By using a SP1/SP3 inhibitor and inhibiting SP1 expression by siRNA, we showed a pivotal role of SP1 in the butyrate and HDACi induction of *TGFB1* expression (Fig. 6 and Supplementary Fig. 3B). Furthermore by mutating the functional SP1 motif in the core promoter of *TGFB1* (TGF_−453/+11SP1_) neither butyrate nor HDACi were able to modulate *TGFB1* expression anymore. This result confirmed that butyrate was enhancing *TGFB1* transcription via SP1 binding on the core promoter. Eventually we confirmed that SP1 binding to *TGFB1* promoter was also essential for bacterial supernatant mediated induction of *TGFB1*.

Recently, Yoshimori’s group demonstrated that *Clostridium butyricum* increases TGFβ1 via a specific mechanism involving Toll-like receptor-2 (TLR2)-dependent signalling and a SMAD auto-induction loop in dendritic cells (DC) present in the *lamina propria*^9^. This TLR2-dependent generation of tolerogenic DC favours regulatory T-cells induction which is similar to what was reported for *Bacteroides fragilis* suggesting a more general mechanism used by several commensal bacteria^60,61^. Kenya Honda’s laboratory also reported that a mixture of *Clostridiales* bacteria from phylogenetic clusters IV and XIVa is sufficient to restore colonic Treg in germ-free mice and this phenotype was dependent on the TGFβ1 produced by IECs^4,21^. Strikingly, the immunosuppressive property of these *Clostridiales* was independent of several known bacterial associated pattern recognition receptors-related signalling pathways such as MYD88 and was related to SCFA production suggesting a mechanism independent to the pathway reported by Yoshimura’s group. From these results, it can be hypothesized that *TGFB1* gene expression can be regulated by cell-type specific transcription factors and signalling pathways.

Here, we describe for the first time the mechanism by which the butyrate-producer of the gut microbiota impacts on the *TGFB1* gene regulation in intestinal epithelial cells that implicates HDACi and the transcription factor SP1. Despite being limited to HT-29 cell-line, we think our results may not be limited to this cell-line. Indeed, previous studies have shown that butyrate activates *TGFB1* expression in diverse cell types including intestinal epithelial cell-lines, such as HCT116 and Caco-2, suggesting that the described mechanism generally impacts IEC^4,53–55^. Therefore, further validation in human primary IECs or intestinal organoids is a future challenge to improve our understanding of the complex impact of butyrate on IECs. Long co-evolution of microbiota and host cells appears to have resulted in multiple mechanisms to increase *TGFB1* expression by IECs and DCs with an overlapping finality, the generation of protective Tregs. Our study supports this hypothesis and contributes to the understanding of the mechanisms developed by the gut microbiota and especially butyrate-producing bacteria to promote local and systemic immune tolerance.

## Materials and Methods

### Cell Culture

The human epithelial cell lines HT-29 was obtained from the American Type Culture Collection (ATCC, Rockville, MD). HT29 cells were grown in RPMI 1640 supplemented with 10% of heat-inactivated fetal calf serum, 2 mM L-glutamine, 50 U/mL penicillin, 50 U/mL streptomycin in a humidified 5% CO_2_ atmosphere at 37 °C. All culture media and supplements were supplied by Sigma-Aldrich. Mycoplasma contamination was regularly tested using MycoAlert (Lonza) and PlasmoTest (Invivogen).

### Plasmids and reporter cell-lines

A 3.2Kb (−1864/+1370 section, TGF_prom_ and a 1.3 Kb (−1362/+11 section, TGF_−1362/+11_) of the human TGFβ1 promoter were cloned into the pGL4.14 (Promega) luciferase plasmid. The TGF_−453/+11_ construct and its derived mutants (TGF_−453/+11SP1_ and TGF_−453/+11AP1_) were a kind gift of C. Weigert and transferred into pGL4.14 backbone^46^. These constructs were used to establishing the stable HT-29-TGF_prom_, HT-29-TGF_−1362/+11_, HT-29-TGF_−453/+11_, HT-29-TGF_−453/+11SP1_ and HT-29-TGF_−453/+11AP1_ reporter cell-lines after antibiotic selection (hygromycin, 600 µg.mL, InvivoGen). HT-29-NFκB cell line was stably transfected with pNiFty2-SEAP (Invivogen) and has been describe previously^25^.

Human *Ffar2* (GPR43) and *Hcar2* (GPR109a) were cloned after EcoRI and XhoI digestion in pCMV-eGFP-N1 vector (Addgene). Oligonucleotides used for amplification of *FFAR2* were *aaaactcgagatgctgccggactggaa* and *aaaagaattcctactctgtagtgaagtccga*. Oligonucleotides used for amplification of *HCAR2* were *aaaactcgagatgaatcggcaccatctgcaggat* and *aaaagaattcttaaggagaggttgggcccaga*.

### Luciferase Reporter and cell viability Assays

For each experiment, cells were seeded at 3.10^4^ cells per well in 96-well plates 24 h prior to incubation with bacterial supernatants or reagents. The cells were stimulated for 24 h with bacterial supernatants, SCFA or controls (TNFα, IL1β, PMA and RPMI) in a total culture volume of 100 μL per well prior to the luciferase assay. The luciferase activity was quantified as relative luminescence units by a microplate reader (Infinite200, Tecan) and the Neolite Luminescence Reporter Assay System (Perkin-Elmer) according to the manufacturer’s instructions. Secreted embryonic alkaline phosphatase (SEAP) was revealed with the Quanti-Blue reagent (Invivogen) using microplate reader (655 nm Infinite 200, Tecan). The *TGFB1* and NF-κB activities were normalized to the controls, i.e., the unstimulated cells or cells in presence of non-inoculated bacteria culture medium. Experiments were performed in triplicates for at least three independent assays. Cell viability was monitored by MTS measurement using the CellTiter 96 Aqueous One solution (Promega) according to the manufacturer’s recommendations.

### Culture of commensal Strains, preparation of bacterial supernatants and SCFA concentration assessment

135 human intestinal commensal bacterial strains which include 111 different species from the in-house INRA-Micalis collection or from DSMZ were grown. Anaerobic culture conditions were done using the Hungate method^62^. Screened species and strains, corresponding growth media, optical densities (OD), short chain fatty acids (SCFAs) concentrations are listed in Supplementary Table 1. Composition of home-made growth media is listed in Supplementary Table 1. Bacterial cultures were cultured to reach the maximum OD and centrifuged at 3,000 g for 10 to 20 min. Cultures were controlled using Gram staining method, aerobic growth test and fresh observation on microscope. Bacterial supernatants were then collected and filtered on a 0.22 μm PES filters and conserved at −80 °C. Non-inoculated bacteria culture medium served as control. Concentrations of SCFAs produced by cultured bacteria were measured by HPLC and gas chromatography as described^63,64^.

### ELISA

HT-29 cells were seeded at 1 × 10^6^ cells per well in 6 well-plates 24 h prior incubation with Serum-free medium and drugs. Supernatants were collected 24 h later and immediately assessed by ELISA following manufacturer instructions using the TGFβ1 DuoSet kit (R&D systems).

### Reagents and cytokines

All agonists, drugs and inhibitors were dissolved in glycerol, DMSO or water following the manufacturer’s recommendations. SCFAs were from Sigma-Aldrich and used in a range of concentrations from 0.5 to 8 mM. GPRs agonists used were: GPR41: 4-CMTB (1 μM, Tocris 4642) and Tiglic acid (1–10 mM, Sigma); GPR43: AR420626 (1 μM, Cayman) and MCPC (1 mM, Sigma); GPR109a: Niacine (1 mM–10 mM, Sigma) and MK1903 (1 μM, Tocris). GPRs sub-unit inhibitors used were: Pertussis toxin (Ptx, 0.2 μg/ml, Sigma) and U73122 (Sigma 10 μM). MCT inhibitors used were: AR-C155858 (0.4 μM, Tocris), p-Chloromercuribenzoate acid (pCMB, 100 μM, Sigma). HDAC inhibitors used were: Trichostatin A (TSA, 1 μM, Sigma), SAHA (5 μM, Sigma). SP1 competitive inhibitor used was the Mithramycin A (0.1 μM) from Sigma. IL1β (10 ng/ml) and TNFα (10 ng/ml) were from Peprotech. Phorbol 12-myristate 13-acetate (PMA, 100 nM) was from Sigma.

### siRNA assays

4.10^5^ HT29- TGF_prom_ cells were seeded in 12-well plates and transient transfections were done on day 2 and day 3 with 2.5 µL of DharmaFECT 1 (Dharmacon) and 25 nM of ON-TARGETplus smart Pool siRNA SP1 (L-026959-00-0005, NM_001251825.1) or Negative Control (D-001810-10-05). On day 4 cells were plated to 2.10^3^ on 96 wells, on day 5 incubation with reagents was done and incubated for an additional 24 h prior luciferase activity measurement.

### Real-Time PCR

HT-29 cells were seeded in 6 well culture plates at densities of 1.10^6^ per well 24 h before stimulation and total RNA was extracted using RNeasy mini-Kit (Qiagen) according to the manufacturer’s recommendations with Dnase I treatment (R&D). cDNA was synthesized from 2 µg of RNA using the High-Capacity cDNA Reverse Transcription Kit (Applied Biosystems) and 100 ng were used to conduct qPCRs on ABI Prism 7700 (Applied Biosystems) or StepOnePlus Real-Time PCR System (ThermoFischer Scientific). The following Taqman Gene expression assay probes were used: *GAPDH* Hs02758991_g1, *GPR43* Hs00271142_s1, *GPR41* Hs02519193_g1, *GPR109a* Hs02341584_s1, *SMCT1* Hs00377618_m1, *and MCT1* Hs01585687_m1. *GAPDH* was used for normalization. Samples were tested in experimental duplicates and at least biological triplicates.

### Promoter analysis

To search for potential transcription factor binding sites on HT-29-TGF_prom_, we used the MatInspector software tool (Genomatix, Munich, Germany) and Jaspar database.

### Statistical analysis and Graphics

Data were analysed using R and RStudio software. PCA analysis was done with prcomp package and correlation matrix was done with Hmisc package. Graphics were produced with ggplot2 package. Statistical test used were two-tailed, T test or Wilcoxon rank test on medians with a confidence level of 95%. P value: *P < 0.05, **P < 0.01, ***P < 0.001, NS: non-significant. Data come from at least 3 independent biological experimentations.

## Electronic supplementary material

Supplementary Table S1

Supplementary Figures S1-S4
