# Supplementary material for: Butyrate produced by gut commensal bacteria activates TGF-beta1 expression through the transcription factor SP1 in human intestinal epithelial cells
Source: Sci Rep. 2018 Jun 27;8:9742. doi: 10.1038/s41598-018-28048-y (PMC6021401; doi:10.1038/s41598-018-28048-y)
Supplement: Supplementary file 2 — Supplementary Figures S1-S4 [file 41598_2018_28048_MOESM2_ESM.pdf]

Butyrate produced by gut commensal bacteria activates *TGF-beta1* expression through the transcription factor SP1 in human intestinal epithelial cells.

Camille Martin-Gallausiaux[1,2], Fabienne Béguet-Crespel[1], Ludovica Marinelli[1,2], Alexandre Jamet[1], Florence Ledue[1], Hervé M. Blottière[1,3] and Nicolas Lapaque[1*].

1. Micalis Institute, INRA, AgroParisTech, Université Paris-Saclay, 78350 Jouy-en-Josas, France;

2. Sorbonne Université, Collège Doctoral, F-75005 Paris, France.

3.MetaGenoPolis, INRA, Université Paris-Saclay, 78350 Jouy en Josas, France.

* Corresponding author: Dr Nicolas Lapaque

INRA-MICALIS UMR1319, Bat 442, Domaine de Vilvert 78350, Jouy-en-Josas, France.

e-mail: nicolas.lapaque@inra.fr

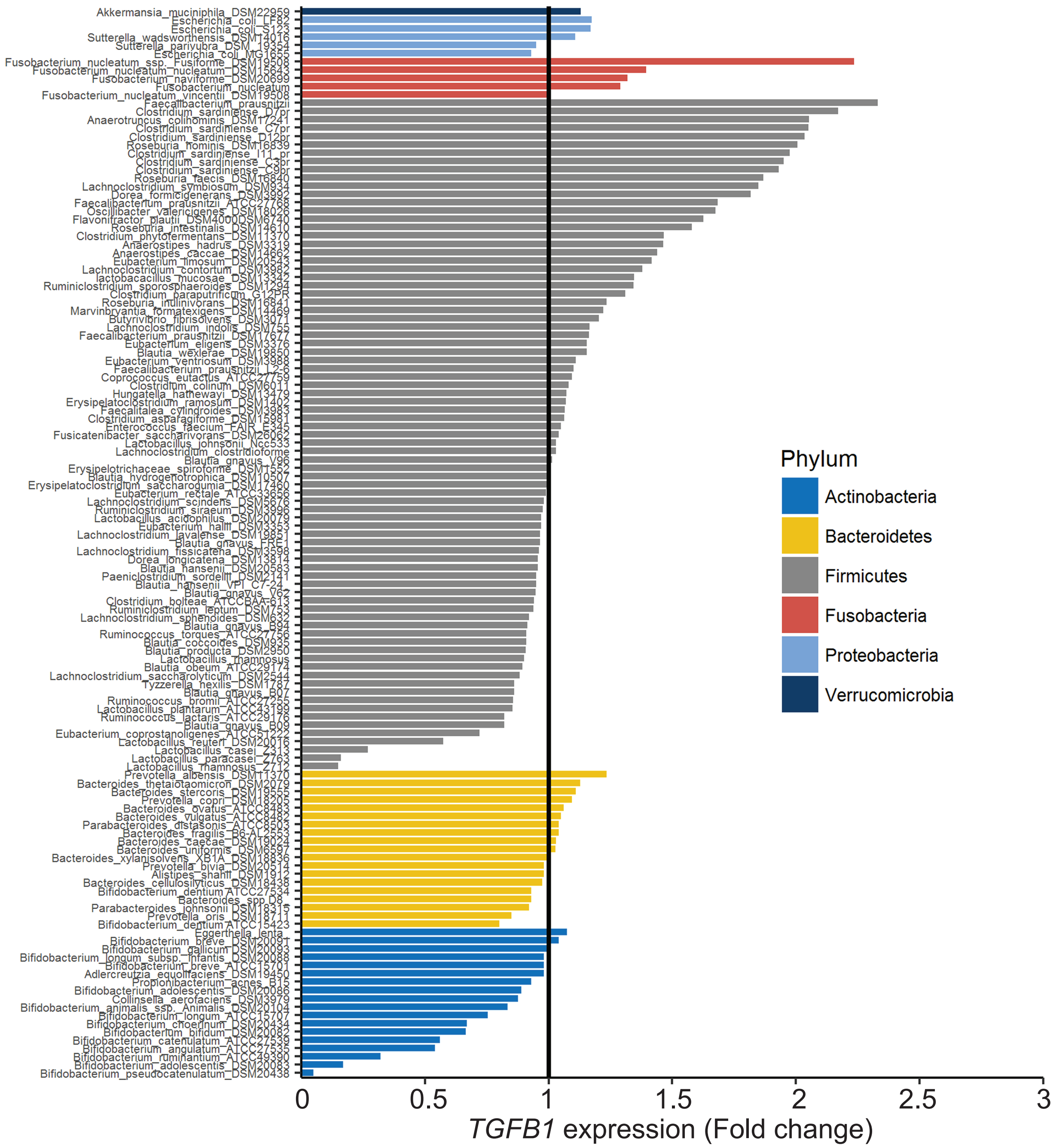

Supplementary Figure S1: Impact of commensal bacteria on *TGFB1* expression in HT-29. Culture supernatants of a wide range of cultivable commensal bacteria were applied on the HT-29-TGF$_{prom}$ reporter system (10% vol/vol). *TGFB1* expression was measured by luciferase activity and expressed as fold change towards its control: bacterial growth medium used in each experiment and for each bacterial culture. Dash lines draw upper and lower zones where *TGFB1 expression* was consider as significantly changed. Actinobacteria in blue, Bacteroidetes in yellow, Firmicutes in grey, Fusobacteria in red and Verrucomicrobiea in light blue.

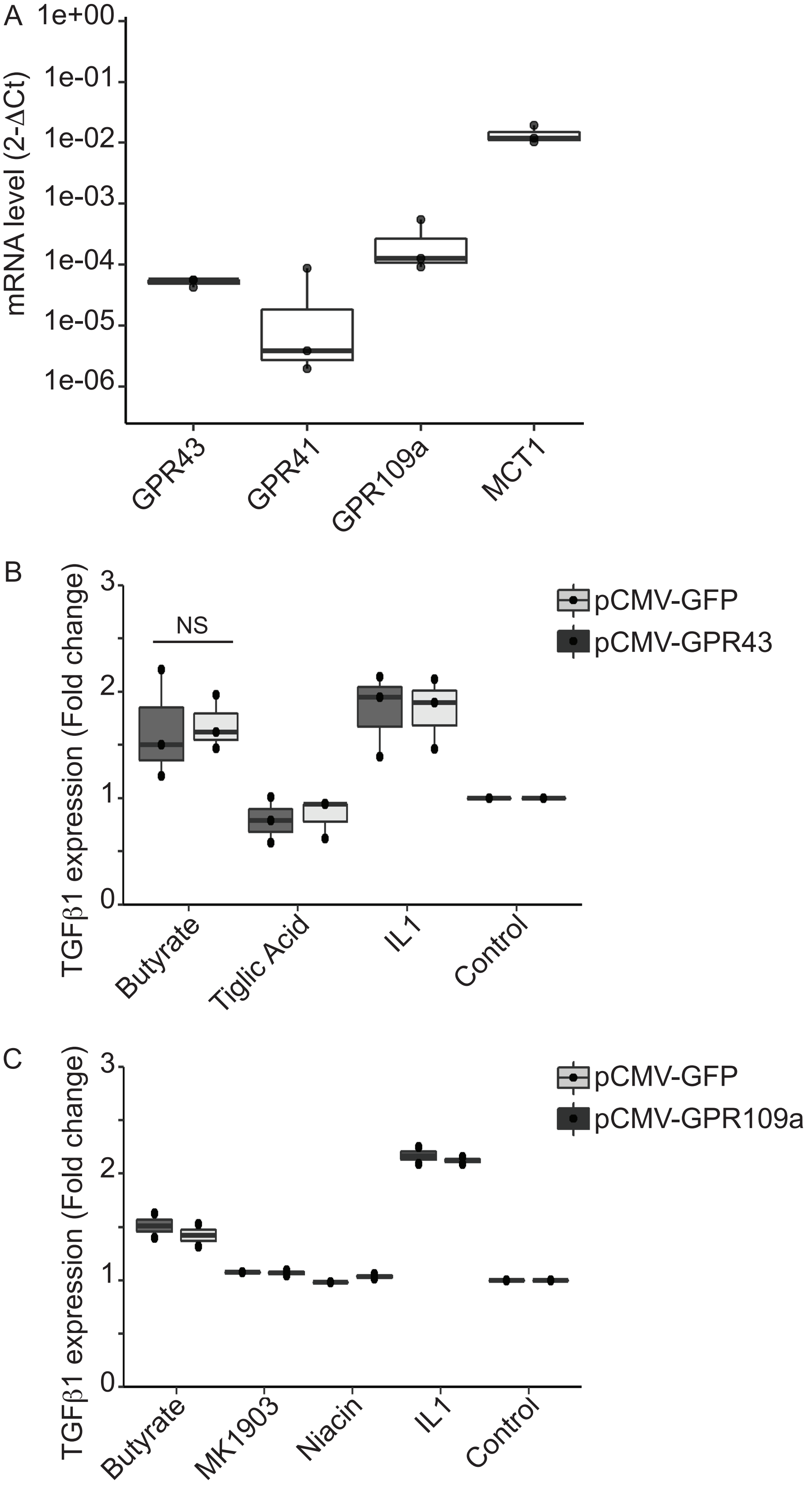

Supplementary Figure S2: Butyrate mediated impact on *TGFB1* is independent of its receptors GPR41, GPR43, GPR109a. (A). Expression of the G-protein coupled receptors *GPR41*, *GPR43*, *GPR109a* and *MCT1* in HT-29. Gene expression was determined by quantitative real-time PCR on total RNA extracted from HT-29. HT-29-TGF_{prom} reporter cells were transfected with pCMV-FFAR2 (GPR43), N=3 (B) and pCMV-HACR2, (GPR109a) N=2 (C) prior to the incubation for 24h with IL1β (10ng/ml), butyrate (2mM) or with the GPR agonists (B) Tiglic acid (10mM) (C): Niacine (10mM) and MK1903 (1µM). *TGFB1* expression was measured by luciferase activity and expressed as median ± quartiles of fold change towards un-stimulated cells, t-test, *P<0.05, **P<0.01, ***P<0.001.

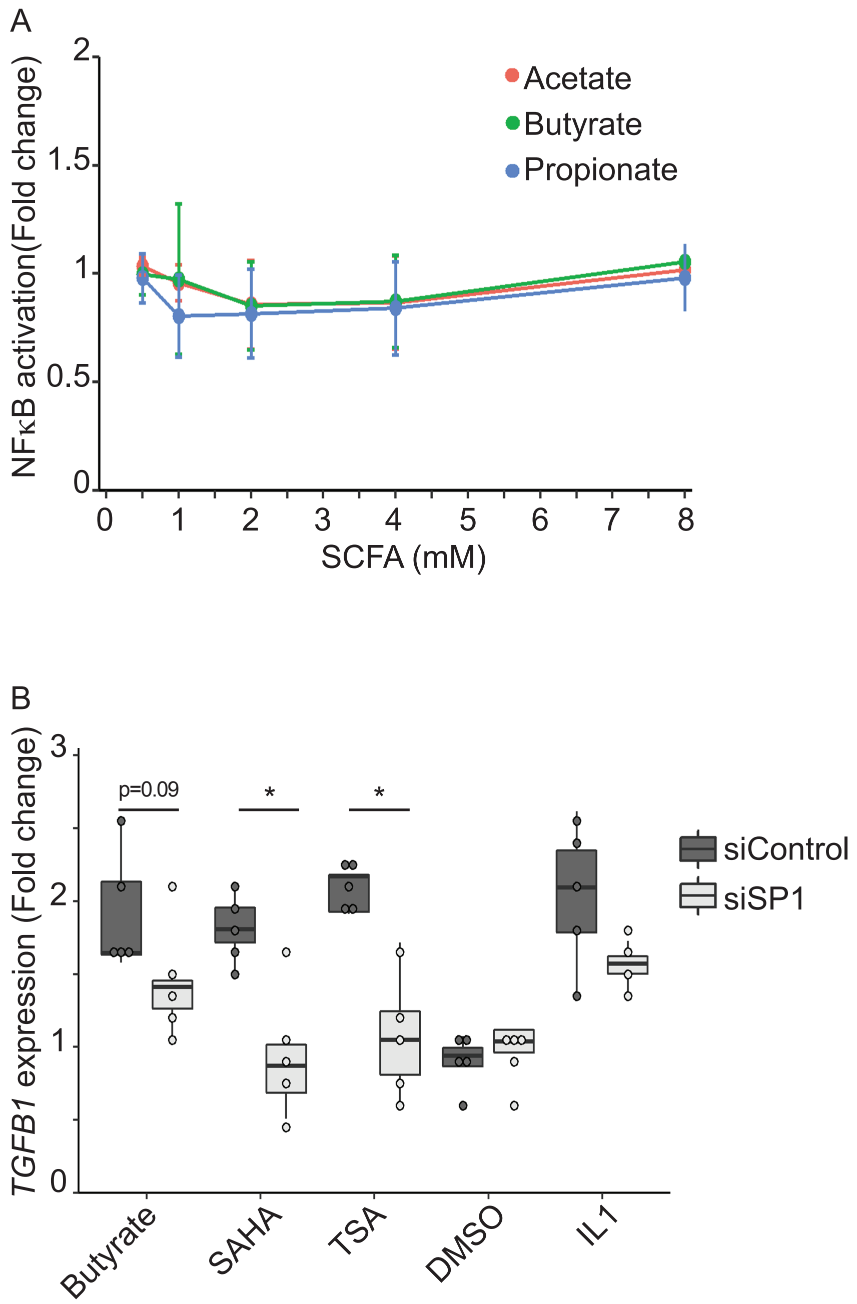

Supplementary Figure S3: (A) HT-29-NFκB reporter cells were incubated for 24h with different concentrations of butyrate. NF-κB activation was measured by SEAP secretion and expressed as fold increase towards un-stimulated cells, N=3. (B) HT-29-TGF_{prom} reporter cells were transfected with SP1 siRNA or control siRNA and incubated with butyrate (2mM), TSA (1µM), SAHA (5µM) or IL1β (100U/ml) for 24h.. *TGFB1* expression was measured by luciferase activity and expressed as median ± quartiles of fold change towards un-stimulated cells. Wilcoxon test, N=5, *P<0.05, **P<0.01, ***P<0.001.

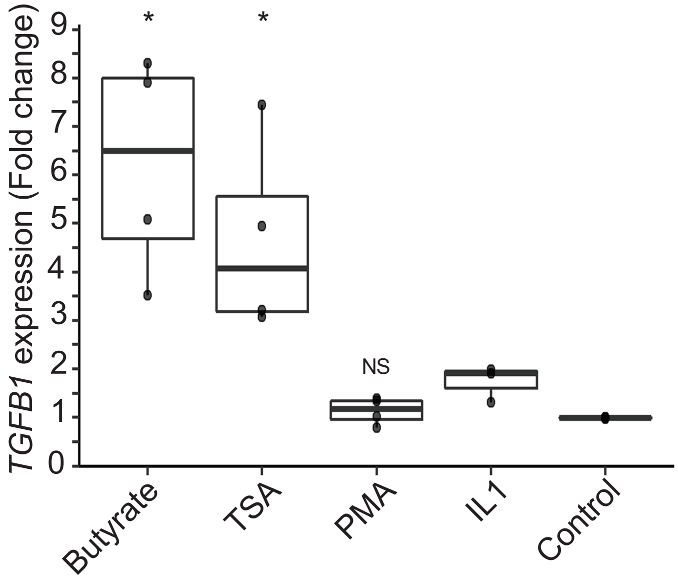

Supplementary Figure S4: HT-29-TGF$_{-453/+11}$ cells with double AP-1 mutations were treated with Butyrate (4mM), TSA (1μM), IL1β (10ng/ml) or PMA (100nM) for 24h. *TGFB1* expression was measured by luciferase activity and expressed as median ± quartiles of fold change towards un-stimulated cells. Wilcoxon test, N=4, *P<0.05, **P<0.01, ***P<0.001.
